# The 12-item World Health Organization Disability Assessment Schedule II (WHO-DAS II): a nonparametric item response analysis

**DOI:** 10.1186/1471-2288-10-45

**Published:** 2010-05-20

**Authors:** Juan V Luciano, José L Ayuso-Mateos, Jaume Aguado, Ana Fernandez, Antoni Serrano-Blanco, Miquel Roca, Josep M Haro

**Affiliations:** 1 Parc Sanitari Sant Joan de Déu, Fundació Sant Joan de Déu, Sant Boi de Llobregat, Barcelona, Spain; 2 Red de Investigación en Actividades Preventivas y Promoción de la Salud, RedIAPP, Barcelona, Spain; 3 Departamento de Psiquiatría, Universidad Autónoma de Madrid, Madrid, Spain; 4 Servicio de Psiquiatría, Hospital Universitario de la Princesa, Madrid, Spain; 5 Centro de Investigación Biomédica en Red en Salud Mental, CIBERSAM, Madrid, Spain; 6 Departament de Salut Pública, Universitat de Barcelona, Barcelona, Spain; 7 Institut Universitari d'Investigació en Ciències de la Salut, Universitat de les Illes Balears & Unidad de Psiquiatría y Psicología Clinica - Hospital Juan March, Palma de Mallorca, Spain

## Abstract

**Background:**

Previous studies have analyzed the psychometric properties of the World Health Organization Disability Assessment Schedule II (WHO-DAS II) using classical omnibus measures of scale quality. These analyses are sample dependent and do not model item responses as a function of the underlying trait level. The main objective of this study was to examine the effectiveness of the WHO-DAS II items and their options in discriminating between changes in the underlying disability level by means of item response analyses. We also explored differential item functioning (DIF) in men and women.

**Methods:**

The participants were 3615 adult general practice patients from 17 regions of Spain, with a first diagnosed major depressive episode. The 12-item WHO-DAS II was administered by the general practitioners during the consultation. We used a non-parametric item response method (Kernel-Smoothing) implemented with the TestGraf software to examine the effectiveness of each item (item characteristic curves) and their options (option characteristic curves) in discriminating between changes in the underliying disability level. We examined composite DIF to know whether women had a higher probability than men of endorsing each item.

**Results:**

Item response analyses indicated that the twelve items forming the WHO-DAS II perform very well. All items were determined to provide good discrimination across varying standardized levels of the trait. The items also had option characteristic curves that showed good discrimination, given that each increasing option became more likely than the previous as a function of increasing trait level. No gender-related DIF was found on any of the items.

**Conclusions:**

All WHO-DAS II items were very good at assessing overall disability. Our results supported the appropriateness of the weights assigned to response option categories and showed an absence of gender differences in item functioning.

## Background

The World Health Organization Disability Assessment Schedule II (WHO-DAS II) was developed by the WHO's Assessment, Classification and Epidemiology Group within the framework of the WHO/NIH Joint Project on Assessment and Classification of Disablements [1]. It is a 36-item instrument designed to measure disability irrespective of health-related etiology in six domains: understanding and communicating, getting around, self-care, getting along with people, life activities, and participation in society. These domains reflect two dimensions of the International Classification of Functioning, Disability and Health (ICF) model [2]: activity limitations (understanding and communicating; getting around; and self-care) and participation (getting along with others; life activities; and participation in society). A one-dimensional screener with 12 items was also developed for measuring global disability, which is especially suitable for epidemiological studies and routine outcome assessment.

The psychometric properties of the WHO-DAS II have been tested in approximately 28 centres in more than 18 geographically and culturally diverse countries. Previous studies, conducted with patients suffering diverse physical and mental conditions, have focused on the factor structure, internal consistency, item-total correlations, etc, using classical test theory as framework [3-10]. However, this type of analyses rely on omnibus statistics that average over levels of individual variation and offer no means to gauge the quality of individual items or options across different levels of disability. Moreover, traditional analyses are sample dependent, showing certain variations among clinical groups and even within clinical groups.

Item response theory (IRT) methods are powerful tools that provide detailed information about item functioning [11-13]. IRT comprises a group of parametric and non-parametric models expressing the probability of a particular response to a scale item as a function of the latent trait of the individual and of characteristics of the item [14]. Some advantages of IRT models with respect to traditional test methods for health outcome assessment are the next [11,15]: (1) item statistics are independent of the individuals included in the sample and person statistics are independent of the administered items (invariance); (2) the standard error of the estimate is unique for each latent trait level; whereas the traditional test-based standard error of the measurement is assumed to be the same for all individuals regardless of their underlying trait level; (3) item information and test information vary as a function of the underlying trait level; (4) it is possible to assess differential item functioning (DIF), defined as different probabilities of endorsing an item by individuals from two groups who are equal on the latent trait. Finally, it is important to highlight that IRT and classical test theory methods have not to be conceived as rivals, in fact, as Pollard et al [16] have recently pointed out, the use of both methods is more informative than only using one of the methods.

The present work extends previous studies using IRT methods to assess the item quality of the 12-item WHO-DAS-II. First, a non parametric item analysis [17,18] was performed to examine the effectiveness of each item and their options in discriminating between changes in the underliying disability level, among patients with a first time diagnosed major depressive episode. Our second specific objective was to evaluate WHO-DAS II items for DIF related to gender.

## Methods

In the present work we utilized the ERASMAP dataset. The ERASMAP was a cross-sectional observational study carried out in Spain. It was designed to identify the sociodemographic and clinical factors associated with diagnostic delay in primary care patients with a first time diagnosed major depressive episode. A total of 1210 general practitioners from 874 healthcare centres agreed to participate in the study, which was performed in accordance with the ethical standards laid down in the 1964 Declaration of Helsinki and was approved by the Clinical Research and Ethics Committee of the University Hospital La Princesa (Madrid, Spain).

### Participants

3615 adult (18 years or older) patients attending general practice, who were for the first time diagnosed with major depressive episode. Patients with a previous diagnosis of major depressive episode, bipolar disorder, schizophrenia or delusional disorder, and those who were receiving treatment with any psychotropic medication were not included in the study.

### Measures

- The *12-item interviewer administered version of the World Health Organization Disability Assessment Schedule II *(12-item WHO-DAS II) [1,8,19]. Respondents are asked to state the level of difficulty experienced taking into consideration how they usually do the activity, including the use of any assistive devices and/or the help of a person. In each item, individuals have to estimate the magnitude of the disability during the previous 30 days using a five-point scale (none = 1, mild = 2, moderate = 3, severe = 4, extreme/cannot do = 5). The total score is calculated with a SPSS syntax (available through the WHO) and may vary from 0 to 100 with higher scores reflecting greater disability (the score indicates the percent of the highest possible score obtained). The Spanish version applied in this study demonstrated sound psychometric properties and evidence of unidimensionality in a previous work [8] (Mean = 53.83, SD = 17.63; Exploratory factor analysis: percentage of variance explained by the 1^st ^factor = 46.15%; ratio of the first to the second eigenvalue > 3; all factor loadings > 0.55; Cronbach's α = 0.89).

### Procedure

During the consultation, the participating general practitioners assessed the patients meeting the inclusion criteria using a paper-and-pencil interview. Prior to the assessment, all patients had provided written informed consent.

### Data analyses

First, we examined item characteristic curves (ICC) for patients to examine each item's overall ability to discriminate among the levels of disability, with steeper slopes indicating a closer relationship to the latent trait and therefore a more discriminating item [20]. The dashed vertical lines in each plot indicate the percentage of individuals that fell below various standard normal scores. The vertical lines of varying length on the ICC are error bars that indicate the estimated 95% point-wise confidence limits for the value of the curve (item score) at a specified disability level (standard normal score). The wider these error bars, the more uncertainty associates with a respondent's item response at that trait level. Second, an item's effectiveness also depends on how well its options function. We examined option characteristic curves (OCC) for each item to check whether each option was dominant over an appropriate but limited range of the trait thus reflecting the rank order of the item options. If OCCs are not distinguishable or if their endorsement probability is never dominant over an appropriate range of the trait, then the original differential weighting of these options is considered inappropriate, and they should be combined or dropped [21].

We decided to use the non-parametric kernel-smoothing technique [22] for examination of item and option characteristics as well as for DIF analysis taking into account the following considerations: non-parametric IRT models do not require complex estimation procedures, can be applied to relatively small data sets, are less imposing concerning distributional form of item response functions and help to avoid misleading results obtained from parametric IRT models. In this work, the analyses were conducted with TestGraf [23]. With the kernel-smoothing technique implemented by TestGraf, the researcher determines the item response functions directly from the data without forcing the data to conform to a logistic IRT model.

DIF is a bias that occurs when a scale item performs differently across different demographic groups after controlling for the underlying trait measured by the instrument. To examine DIF, TestGraf calculates a weighted average of the squared difference between the focal or comparison group's probability of endorsing an item and the reference or baseline group's probability of endorsing an item. Thus, a composite index of DIF is obtained after comparison of the response characteristic curves. If the focal group (the women in our case) has a higher probability of endorsing an item, the index of DIF will be positive, while the index will be negative if the reference group (the men in our case) has a higher probability. Taking into account the Santor and colleagues' criteria [24], a value equal or greater than 0.30 indicates the existence of DIF, whereas a value equal or less than 0.10 indicates little or no DIF.

## Results

Patient characteristics are displayed in Table 1. We used means and standard deviations for continuous variables and percentages for categorical variables.

**Table 1:** Characteristics of the study sample (n = 3615)

Sociodemographic variables	
***Gender %***	
*Male*	32.67
*Female*	67.33
***Age, M (SD)***	50.01 (13.89)
***Marital status %***	
*Married*	54.67
*Living with a partner*	9.48
*Single*	11.76
*Separated/divorced*	12.91
*Widowed*	11.18
***Education level %***	
*Did not graduate from primary school*	21.44
*Primary school*	15.12
*Secondary school*	43.14
*University*	20.30
***Work status %***	
*Student*	1.31
*Homemaker*	23.36
*Paid employment*	57.70
*Paid employment but in sick leave*	39.52
*Unemployed*	2.34
*Permanent disability*	1.78
*Retired/pensioner*	13.37
*Others*	0.14

### Items effectiveness

**Figure 1:**
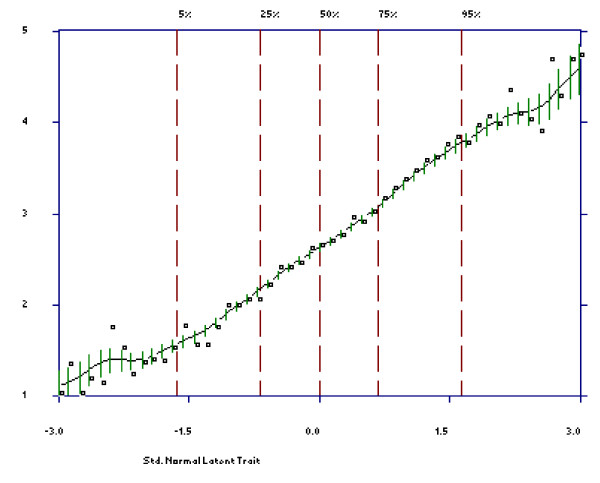
**Item Characteristic Curve for item 1**.

**Figure 2:**
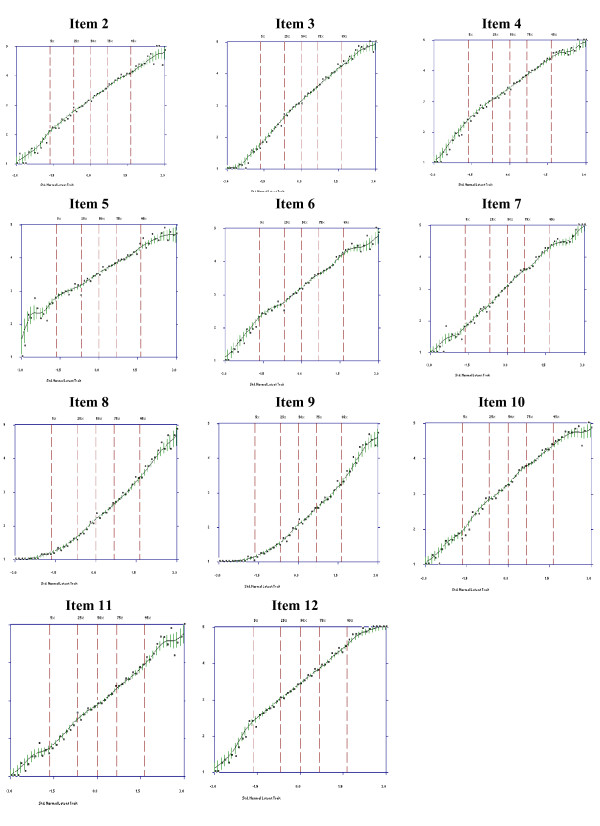
**Item Characteristic Curves for items 2-12**.

As can be seen in Figures 1 and 2, all items discriminated well over the whole trait range since all ICCs increased steadily with increasing trait level. The ascending slope curves indicate that those patients with higher expected total scores were increasingly likely to report higher item scores. Wider error bars are observed for respondents with expected scores at the bottom and the top 5% of the trait level, reflecting poorer prediction at these trait levels. In Figure 1 the ICC of item 1 is displayed for illustrative purposes, whereas the ICCs for the other items are displayed in Figure 2.

### Options effectiveness

**Figure 3:**
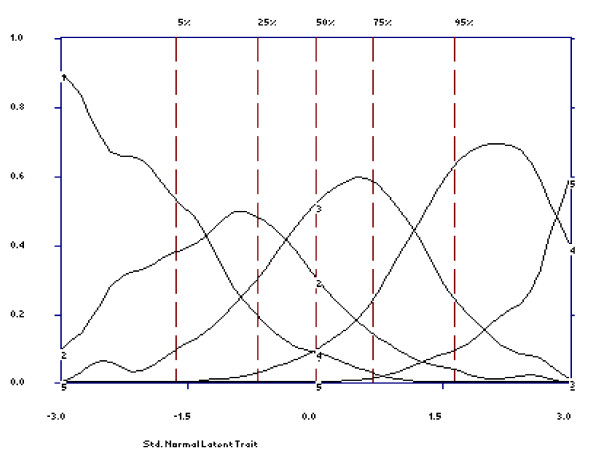
**Option Characteristic Curve for item 1**.

**Figure 4:**
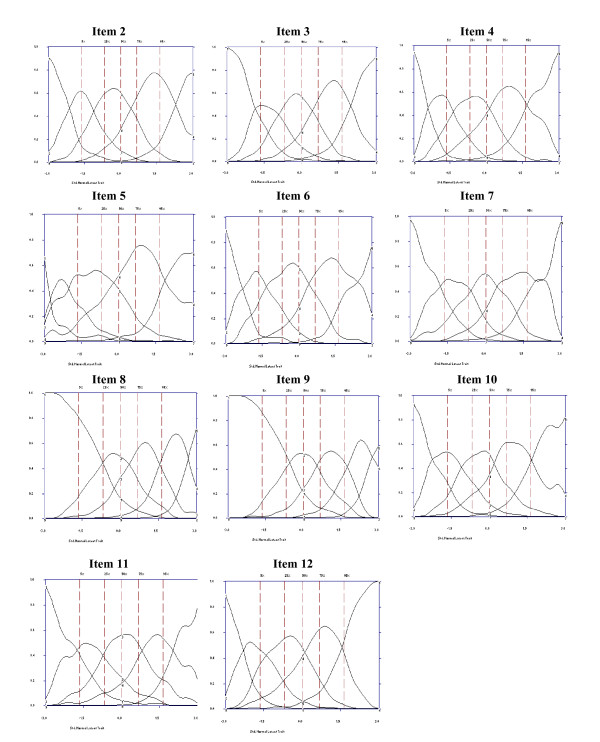
**Option Characteristic Curves for items 2-12**.

As is shown in Figures 3 and 4, all items were effective in making discriminations throughout levels of latent disability. The examination of the OCCs clearly indicated that each increasing option became more likely than the previous option as levels of disability increased. All five options were being used with each option dominating the response probability over an appropriate but limited range of the trait level. For instance, looking at the OCCs for item 1 (see Figure 3), we observe that the probability of choosing option 1 (none) was high (between 0.57 and 0.90) for low disability respondents (bottom 5% of the trait level) and dropped off steeply with increasing disability levels, approaching a near zero probability of endorsement for the highest levels of the trait. The probability of endorsing options 2 (mild), 3 (moderate), 4 (severe), peaked at a standard normal score between -1.5 and -0.5, -0.5 and 1.0, 1.5 and 2, respectively. Finally, the option 5 (extreme/cannot do) was high (between 0.50 and 0.60) for extremely disabled respondents (about top 2-3% of the trait level). The OCCs for items 2-12 are displayed in Figure 4.

### Differential Item Functioning (DIF) related to gender

Prior to the DIF analysis, we conducted a Student's t-test for independent samples in order to examine if there were significant gender differences in disability at the scale level. The analysis revealed that women (n = 2360; M = 54.96, SD = 17.52) had significantly higher disability than men (n = 1125; M = 51.54, SD = 17.69), t_(3483) _= 5.36, *p *< .001.

Then, the men were arbitrarily chosen as the baseline group, while the women were part of the focal group in the DIF analysis. Although the composite DIF index was positive in all items, it indicated an absence of substantially meaningful gender-related differences (M = .084, SD = .062; Min = .008; Max = .188). In other words, scores from the items do not overestimate the level of disability in women compared with men. In Table 2, we show mean raw scores for men and women, along with mean gender item bias for each of the items on the WHO-DAS II.

**Table 2:** Mean raw scores and mean gender item bias for men and women.

Items	Men	Women	Bias
1. Standing for long periods such as 30 minutes?	2.51	2.71**	.138
2. Taking care of your household responsibilities?	2.93	3.29**	.098
3. Learning a new task, for example, learning how to get to a new place?	2.94	3.18**	.108
4. How much of a problem did you have in joining in community activities (for example, festivities, religious or other activities) in the same way as anyone else can?	3.40	3.46*	.029
5. How much have you been emotionally affected by your health problems?	3.48	3.54	.011
6. Concentrating on doing something for ten minutes?	3.17	3.23	.008
7. Walking a long distance such as a kilometre [or equivalent]?	2.90	3.16**	.077
8. Washing your whole body?	2.18	2.25*	.186
9. Getting dressed?	2.07	2.15*	.188
10. Dealing with people you do not know?	3.24	3.29	.055
11. Maintaining a friendship?	2.91	2.89	.069
12. Your day to day work?	3.43	3.46	.036

*Note*: Bias refers to bias in expected item scores. Tests of significance indicate differences between men and women.

*p < 0.05, **p < 0.001.

## Discussion

In the present work, we employed a non parametric IRT method to examine the items effectiveness, response category functioning and differential item functioning related to gender of the 12-item WHO-DAS II.

Overall, the results obtained in the present research indicate that all WHO-DAS II items performed well at discriminating varying levels of disability. The inspection of the ICCs and the OCCs indicated that all items assessed well the entire continuum of disability. We could see that certain items, for instance items 8 and 9 (bathing and dressing, respectively), ask about activities in which patients clearly experience fewer problems, whereas in others, for instance items 5 and 12 (emotionally affected by the health problem and work, respectively), patients report more problems or difficulties. Finally, the weights assigned to the individual item options are appropriate for measuring the underlying trait due to the absence of overlap between adjacent OCCs.

Following Santor et al criteria [25], we can consider the items as "good" or "very good" because there was some range of severity in which the majority of options were more likely to be endorsed than any other, the OCCs increased rapidly with changes in overall severity, the region in which each option was most likely to be endorsed were ordered, left to right, in accordance with their option scores (weights), and options spanned the full continuum of severity from -3 to +3. In addition, we did not find "easy" (the majority of options on an item are endorsed at low levels of severity) or "hard" items (the majority of options on an item are endorsed at high levels of severity). Finally, men and women who were at the same point on the disability continuum did not respond differently to items on the WHO-DAS II, that is, group mean differences between men and women in disability cannot be attributable to gender item bias.

Our results partially support those obtained by Rehm and collaborators [26] with a previous version of the instrument. Using data from two field trials, these authors examined the 12-item WHO-DAS II (screener version that possessed different items) by means of confirmatory factor analysis, non-parametric (Mokken scale analysis) and parametric (Birnbaum's two-parametric model) methods of IRT. The non-parametric analysis indicated that the scale could be considered of medium scalability, however, the parametric analysis indicated that the ICCs did intersect, suggesting the need of developing a new version of the instrument that would be IRT compatible.

We have to acknowledge the following limitation in the present work. Although the 12-item WHO-DAS II is sufficiently unidimensional for IRT analysis, we found covariation in a previous study [8] within some pairs of items (1 and 7, 8 and 9, 10 and 11; the confirmatory factor analysis indicated that the three error covariances were statistically significant, ranging from a minimum of 0.42 to a maximum of 0.83), which supposes a violation of the local independence criterion required by IRT. However, having in mind that IRT methods are quite robust to minor violations related to the local independence assumption, especially when a scale consists of 10 or more ítems [20], we think that there are not enough reasons to judge the results reported above as unreliable or non valid.

## Conclusions

IRT methods enable researchers to examine important scale properties that can not be addressed with traditional analyses [27]. In this work, using non parametric IRT analyses, we found that WHO-DAS II items and options discriminate well among different latent levels of disablement and that it is a nonbiased instrument with respect to gender. Future studies should try to extend our findings using a parametric IRT model in order to confirm that the instrument is IRT compatible and allows cross-cultural comparability.

## Competing interests

JLA-M, MR and JMH received economic compensation from H. Lundbeck A.S for the design of the study. The other authors declare that they have no competing interests.

## Authors' contributions

JLA-M, MR and JMH are the principal investigators, developed the original idea for the research and made the study design. JVL managed the literature searches, undertook the statistical analyses and wrote the first draft of the manuscript with the collaboration of JA, AF and ASB. All authors revised the manuscript and approved its final version.

## Pre-publication history

The pre-publication history for this paper can be accessed here:

http://www.biomedcentral.com/1471-2288/10/45/prepub
